# Supplementary material for: Comparative analysis of mental and physical fatigue on motor coordination, visual search patterns, perceived effort, and performance outcomes in closed-loop aiming task
Source: PLoS One. 2025 Jul 18;20(7):e0324063. doi: 10.1371/journal.pone.0324063 (PMC12274008; doi:10.1371/journal.pone.0324063)
Supplement: Attachment — Submitted filename: Reply to the referees.docx [file pone.0324063.s003.docx]

**Reviewer #1 Comments:**

1. **Abstract:**
   - **Recommendation:** “I recommend that abbreviations be first listed in parentheses after they have been mentioned descriptively.”
   - **Action:** The referee's valuable insight was applied.
2. **Participants:**
   - **Recommendation:** “I recommend mentioning the mean age and SD, gender, and level of golf practice.”
3. **Action:** The referee's valuable insight was applied.
4. **Results:**
   - **Recommendation:** “I recommend revising, -Results are that..., possibly The recorded results reveal ..., or The results show that.....”
   - **Action:** The referee's valuable insight was applied.
   - **Keywords:**
   - **Recommendation:** “I recommend adding a new word that refers to the sport referred to.”
   - **Action:** Golf performance was added as a keyword.
5. **Introduction:**
   - **Recommendation:** “I recommend, at the end of the section, mentioning the objectives or hypotheses of this study.”
   - **Action:** We added the required section to the manuscript.
6. **Methods:**
   - **Recommendation:** “I recommend introducing a new subsection called Study design where the typology of the study and other specific aspects should be mentioned.”
   - **Action:** We added a new subsection titled “Study Design and we combined the Procedure part to Study Design.
7. **Ethical Approval:**
   - **Recommendation:** “I recommend mentioning whether the consent of the subjects to participate in the study was obtained.”
   - **Action:** We added the following content: “Informed consent was obtained from all participants prior to their participation.”
8. **Methods Subsections Numbering:**
   - **Recommendation:** “I recommend that the subsections in the Methods section be numbered.”
   - **Action:** The referee's valuable insight was applied.
9. **Discussion Section:**
   - **Recommendation:** “Lines 359-368 should be moved to the end of the Discussion section.”
   - **Action:** edited.
10. **Practical Implications and Future Directions:**
    - **Recommendation:** “I recommend moving to the Discussion section.”
    - **Action:** edited.
11. **Conclusions:**
    - **Recommendation:** “In the conclusions section, only the main conclusions focused on the results should remain.”
    - **Action:**… edited

**Reviewer #2 Comments:**

1. **Figures:**
   - **Recommendation:** “The quality of the figures is poor and they are not legible, and it is not clear what is shown due to the authors not labelling the y-axis.”
   - **Action:** The Y label was added. We used the TIF format, which gives unlimited resolution. The problems may come from the PDF file. Therefore, the referee can download the docx file from the link in the PDF file.
2. **Citations Format:**
   - **Recommendation:** “The citations are a mixture of numbers and author+year format without following the journal format.”
   - Action: edited
3. **Exclusion Criteria:**
   - **Recommendation:** “The exclusion criteria, ‘any unwillingness to cooperate throughout the study,’ calls into question the voluntariness of participant involvement, a fundamental requirement stipulated by the Declaration of Helsinki.”
   - **Action:** edited.

**Reviewer #3 Comments:**

1. **Abstract:**
   - **Recommendation:** “we recommend including in the Results some relevant results identified in the study, currently only the textual interpretation of the results appears.”
   - **Action:** Abstract results rewritten.
2. **Introduction:**
   - **Recommendation:** “To detail more specifically the novel aspects of the present study in relation to previous studies on the same topic.”
   - **Action:** we added this missed part to the manuscript's content.
3. **Hypothesis:**
   - **Recommendation:** “Formulate the hypothesis of the study.”
   - **Action:** We added the required section to the manuscript.
4. **Study Design:**
   - **Recommendation:** “To detail the periodization of the study: year, testing periods, stages of the study, etc
   - **Action:** In the participant section, we mentioned that the date range for participant recruitment was June 15, 2023, to September 10, 2023. In the methods section, we divided the study into three phases, including: The experiment was carried out in three phases for each participant: (1) a pre-fatigue baseline session, (2) a fatigue induction session (mental or physical), and (3) a post-fatigue session.
5. **Discussion:**
   - **Recommendation:** “- To add at the end of the Discussions - The practical implications of the study based on the relevant results.
   - - To delimit the limits of the study
   - **Action:** We have rewritten this part.
6. **Conclusions:**
   - **Recommendation:** “We recommend restructuring the conclusions in relation to the main results identified in the study. It is not normal for bibliographical references to appear in the conclusions section. Conclusions refer to the relevant aspects of the present study and do not make connections to previous studies. Sentences where reference is made to previous studies should be moved to the Discussion section.
   - - Future research directions in accordance with the topic of the study should be mentioned.”
   - **Action:** edited
